# Endocrine therapy for early breast cancer in the era of oral selective estrogen receptor degraders: challenges and future perspectives

**DOI:** 10.1097/CCO.0000000000001085

**Published:** 2024-08-12

**Authors:** Liliana Ascione, Grazia Castellano, Giuseppe Curigliano, Paola Zagami

**Affiliations:** a Division of Early Drug Development for Innovative Therapies, European Institute of Oncology (IEO) IRCCS; b Department of Oncology and Hematology (DIPO), University of Milan, Milan, Italy

**Keywords:** breast cancer, curative treatment, endocrine therapy, selective estrogen receptor degraders

## Abstract

**Purpose of review:**

Growth and survival of hormone receptor positive breast cancer cells are dependent on circulating hormones (e.g., estrogen and progesterone). Endocrine therapy improved outcomes in both early and advanced hormone receptor positive breast cancer. These treatments include drugs with different mechanisms of action, namely selective estrogen receptor modulators (SERM), aromatase inhibitors, and selective estrogen receptor degraders (SERDs). SERDs represent estrogen receptor antagonists, favoring its degradation and thus interfering with proliferation genes transcription and activation. Fulvestrant is the first approved SERD, administered intramuscularly for treating advanced breast cancer.

**Recent findings:**

Oral SERDs have been tested to overcome the limitation of the intramuscular administration, and to increase SERD bioavailability. Recently, an oral SERD, Elacestrant, has been approved by the Food and Drug Administration (FDA) for patients carrying an *ESR1* mutation. In fact, oral SERDs seem to be effective in tumors harboring ESR1 mutations, a well known mechanism of resistance to endocrine therapy (especially aromatase inhibitors).

**Summary:**

More recently, oral SERDs have been tested in patients with early hormone receptor positive breast cancer, although their impact on survival and in this curative setting compared to standard endocrine therapy still needs to be elucidated. The best timing and duration of SERD administration and specific biomarkers in (neo)adjuvant setting remain largely unknown.

## INTRODUCTION

Breast cancer is the most frequently diagnosed tumor and the leading cause of cancer death in women [1,2]. More than 70% of these are hormone receptor positive (HR+) breast cancers [3^▪▪^]. 

**Box 1:**
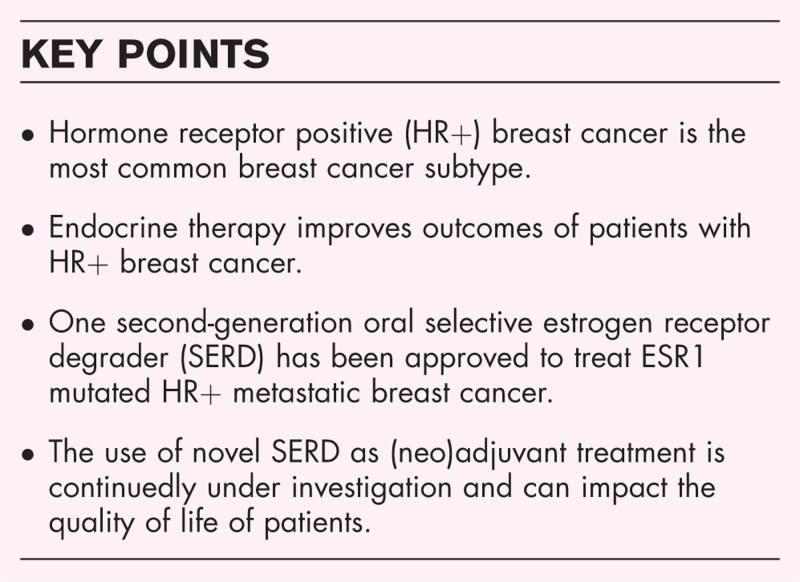
no caption available

**FIGURE 1:**
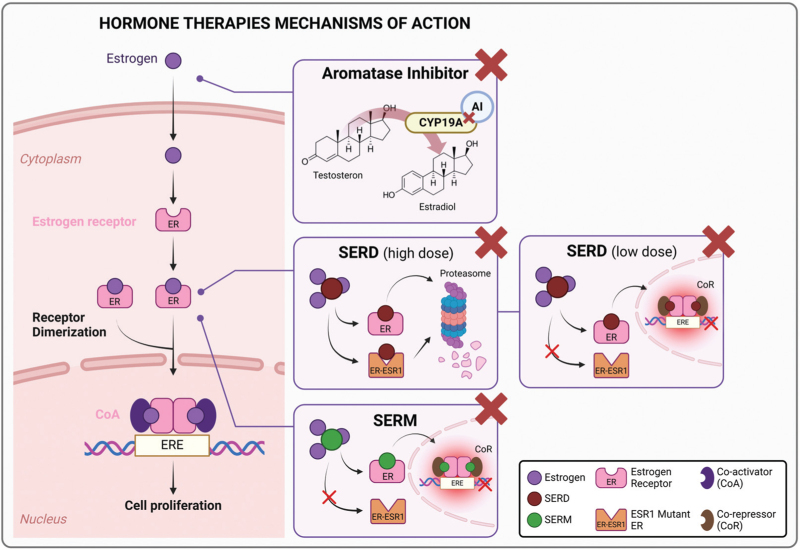
Mechanisms of action of hormone therapies. Estrogen receptors (ERs) are steroid receptors acting as transcription factors regulating the expression of certain target genes. Two different ERs can be bound by estrogen: ERα is encoded by the ESR1 gene (chromosome 6), while ERβ is encoded by the ESR2 gene (chromosome 14), both receptors are expressed on normal mammary gland, however ERα is mainly enrolled in promoting breast tumorigenesis, while ERβ is typically downregulated during carcinogenesis. Estrogen receptor is activated through the intracytoplasmic bounding with estrogen, leading to dimerization in cytoplasm moving to nucleus with activation of the estrogen response element (ERE) that are estrogen-responsive genes by binding to a specific DNA sequence. (1) Aromatase inhibitors (e.g., letrozole) block the estrogen biosynthesis inhibiting the enzymatic activity of the estrogen synthase (i.e., aromatase, CYP19) that transforms testosterone in estradiol; (2) SERD (e.g., fulvestrant) antagonizes both ERα and ERβ by favoring its proteasomal degradation and hindering the recruitment of co-activators and the translocation of ER-dimers into the nucleus; SERM modulates (e.g., tamoxifen) compete with estrogens for binding to the estrogen receptor functions. AIs, aromatase inhibitors; ER, estrogen receptors; ERE, estrogen response element; ESR1, mutated-Estrogen Receptor 1 gene; HR, hormone therapies; SERD, selective estrogen receptor degrader; SERM, selective estrogen modulators.

The proliferation and survival of hormone receptor positive breast cancer cells depend on the estrogen receptor pathway that regulates their transcriptional processes (Fig. 1) [4]. Endocrine therapy represents the main therapeutic backbone of hormone receptor positive breast cancer, improving outcomes of the patients in both early and advanced settings [5]. Endocrine therapy agents available to treat hormone receptor positive breast cancer in clinical practice are commonly divided into three groups based on different mechanisms of action: selective estrogen receptor modulators (SERM), steroidal and nonsteroidal aromatase inhibitors, and selective estrogen receptor degraders (SERDs). These drugs can be used alone or in combination with other anticancer agents (e.g., cyclin-dependent kinase 4 and 6 inhibitors - CDK4/6 inhibitors, mTOR inhibitors, PIK3CA inhibitors), depending on the specific therapeutic setting [5].

Most breast cancers are diagnosed in the early setting [6]; however, the rate of distant recurrence is still high [7,8]. Hence, reducing the risk of recurrence through new (neo)adjuvant therapeutic strategies is crucial.

Currently, according to international guidelines, SERM (like tamoxifen) and aromatase inhibitors (letrozole, anastrozole, and exemestane) are included in the treatment landscape of hormone receptor positive early breast cancer (EBC). Specifically, adjuvant endocrine therapies include tamoxifen or aromatase inhibitors with or without ovarian function suppression (OFS) based on the risk of recurrence, menopausal status, and comorbidities. In postmenopausal patients, aromatase inhibitors represent the main therapeutic choice [3^▪▪^]. Escalation strategies in patients with high-risk tumors include the use of (neo)adjuvant chemotherapy and/or the addition of CDK4/6 inhibitors to adjuvant endocrine therapy [9^▪▪^,^10^] or the use of adjuvant Olaparib in germline BRCA mutant carriers [11] Neoadjuvant endocrine therapy is mainly reserved for patients considered chemotherapy unfit due to comorbidities and/or patients with tumors characterized by low-risk features [12^▪▪^]. No SERD agent is approved for EBC treatment.

Fulvestrant has been the first SERD approved for treating metastatic hormone receptor positive breast cancer [13]. In the last years, second-generation SERDs, administered orally, have been developed to overcome the limitation of intramuscular injection and the poor bioavailability related to fulvestrant. This novel drug class is largely investigated in metastatic breast cancer, with promising results, especially in *ESR1* mutant breast cancer, a common mechanism of resistance to endocrine therapy [14,15]. Recently, according to the results of the EMERALD trial, the oral SERD elacestrant has been approved by the Food and Drug Administration (FDA) for treating metastatic breast cancer progressing to at least one line of endocrine therapy, including one containing a CDK4/6 inhibitor, and carrying an *ESR1* mutation. Data coming from trials in the metastatic setting paved the way for new trials investigating oral SERDs in early breast cancer. This review focuses on evidence of oral SERDs in early breast cancer and provides an overview of the ongoing trials (Table 1).

**Table 1:** Select ongoing phase III trials with oral selective estrogen receptor degraders in the adjuvant setting

Drug	Trial (NCT n.)	*N*	Prior ET required	Population	BC stage	Control arm	Adjuvant CDK4/6i or PARPi allowed
Giredestrant	LIDERA (NCT04961996)	4100	≤12w prior ET	Women (pre, peri, post); men	-pN0 and >pT1c + G3 or ki67≥20% or Oncotype /MammaPrint high -pN1, pN2, pN3	Tamoxifen or AI	No
Imlunestrant	EMBER-4 (NCT05514054)	6000	2–5y prior ET	Women (pre, peri, post); men	-pN0 and tumor size ≥5 cm or G3 tumor size 2–5 cm -pN1 and tumor size ≥5 cm or G3 or G2 tumor size 2–5 cm -pN2, pN3	Tamoxifen or AI	Yes
Camizestrant	CAMBRIA-1 (NCT05774951)	4300	2–5y prior ET	Women (pre, peri, post); men	-pT1c-T2, pN0 and G3 or high-risk gene expression assay or Ki-67 ≥20% or prior chemotherapy -pT1c-T2, pN0mic or pN1 and G3 or high-risk gene expression assay or Ki-67 ≥20% -Any pT if ≥2 lymph nodes involved -≥T3 (T4d excluded) regardless nodal status	Tamoxifen or AI	Yes
	CAMBRIA-2 (NCT05952557)	5500	≤12w prior ET	Women (pre, peri, post); men	-pT1c-T3, pN0 or 1 positive lymph node (or only micrometastatic involvement) and G3 or high-risk gene expression assay or Ki-67 >20% or prior chemotherapy -Any T with ≥2 ipsilateral lymph nodes involved -Any T4 tumors regardless of nodal status	Tamoxifen or any AI	Yes
Elacestrant	TREAT ctDNA (NCT05512364)	220	2–7y prior ET and ctDNA+	Women (pre, peri, post); men	-Stage IIB or stage III and completion of adjuvant CT or at least 4 cycles of neoadjuvant CT and residual disease at surgery (≥ypT1c or ypN+)	Tamoxifen or AI	Yes

AI, aromatase inhibitor; BC, breast cancer; CDK4/6i, cyclin-dependent kinase 4/6 inhibitors; CT, chemotherapy; ctDNA, circulating tumor DNA; ET, endocrine therapy; *N*, number of patients; PARPi, Poly ADP-ribose polymerase inhibitors; TBC, to be confirmed; W, weeks; Y, years.

## ORAL SELECTIVE ESTROGEN RECEPTOR DEGRADER MECHANISM OF ACTION AND SAFETY PROFILE

Endocrine therapies have different mechanisms of action, as shown in Fig. 1.

Differently from tamoxifen, SERDs play mainly an antagonist action on estrogen receptor. SERDs directly inhibit the α isoform of the estrogen receptor (ERα), inducing a conformational change that limits its dimerization while promoting its degradation via the proteasome system, thus reducing translocation to the nucleus and the transcription of proliferation genes [16]. Two main SERD types have been tested in clinical trials. SERDs with acrylic acid side chains, GDC-0810 (brilanestrant), AZD9496, and LSZ102, showed low efficacy in performing a full antagonism activity with an unfavorable toxicity profile, thus their investigation was stopped at the early phase of development [17]. On the contrary, SERDs with amino side chain demonstrated better clinical activity, reducing tumor cell proliferation through an improved estrogen receptor antagonization [18], associated with more manageable toxicities; this class includes amcenestrant, camizestrant, elacestrant, giredestrant and imlunestrant [17]. The main toxicity concerns related to oral SERDs are represented by gastrointestinal adverse events, particularly diarrhea, and nausea, along with fatigue. Of note, camizestrant and giredestrant have also been associated with bradycardia in phase I/II trials. Camizestrant also caused ocular toxicities, mostly grade 1 and grade 2 [19^▪^].

## THE ROLE OF SELECTIVE ESTROGEN RECEPTOR DEGRADERS IN THE HORMONE RECEPTOR POSITIVE EARLY BREAST CANCER

Endocrine therapy is the therapeutic pillar for the treatment of hormone receptor positive breast cancer; however, the occurrence of primary and secondary resistance might limit its efficacy over time, representing the main issue to be addressed to improve disease control. The most common and studied mechanism of endocrine resistance is the emergence of genetic mutation(s) involving the *ESR1* gene, which encodes for the estrogen receptor ligand-binding domain (ERα).

This alteration causes a conformational change that constitutively activates estrogen receptor, independently from estrogen binding: such conformational change reduces endocrine therapy affinity, also causing the recruitment of transcriptional coactivators and leading to constitutive transcriptional activity [20].

ESR1 mutations are common in metastatic breast cancer, found in 30–50% of tumors progressed on aromatase inhibitors treatment, while these mutations are rare in endocrine therapy naive primary tumors. SERDs demonstrated higher clinical benefit in *ESR1*-mutated MBC, however, restricting the investigation of oral SERDs to *ESR1* mutation carriers in the early setting may significantly limit their therapeutic potential [21,22].

Since about 30% of patients with high-risk EBC receiving adjuvant endocrine therapy will recur and develop metastatic disease, it should be assumed that also other mechanisms of resistance to endocrine therapy can be involved. Moreover, patients in the curative setting frequently complain of many adverse events related to adjuvant endocrine therapy, which often leads to its discontinuation before completing the 5 years.

In this context, SERDs might play a role in the early setting as (neo)adjuvant treatments.

### Oral selective estrogen receptor degrader in the adjuvant setting

Clinical trials investigating oral SERDs in the neoadjuvant setting are focused mainly on postmenopausal patients [23], while recent adjuvant trials have been specifically designed to include both pre and perimenopausal women and men [10].

Adjuvant giredestrant has been evaluated in the LIDERA trial. The study randomizes 4100 patients, whether pre, peri, or postmenopausal women with high-risk EBC, as well as men who have undergone prior surgery with curative intent to receive giredestrant at 30 mg *quaque die* or physician's choice adjuvant endocrine monotherapy, which may include tamoxifen or an aromatase inhibitor. Notably, participants in this trial are capped at receiving a maximum of 12 weeks of endocrine therapy. Stratification factors encompass the patient's risk profile (defined by various factors), geographic regions, prior chemotherapy, and menopausal status. Recruitment has concluded, and the results are awaited [24^▪^].

First data on imlunestrant came from the phase 1a/b EMBER trial in pretreated MBC. During the dose escalation phase, patients were assigned to imlunestrant alone, imlunestrant in combination with everolimus, or imlunestrant in combination with alpelisib. Nearly 50% of the patients in the imlunestrant arm had an *ESR1* mutation. The trial results indicated a median progression-free survival (mPFS) of approximately 4 and 5 months for the overall and ESR1-mutated population respectively, treated with monotherapy [25,26].

Based on these results, the EMBER-4 trial was designed to investigate imlunestrant in the adjuvant setting. The study will randomize about 6000 patients to imlunestrant at the standard dose of 400 mg daily (q.d.) or physician's choice endocrine therapy, which may involve tamoxifen or an aromatase inhibitor. Unlike previous trials, patients in this study should have received at least 2 years of endocrine therapy, extending up to 5 years in the adjuvant setting. Stratification factors include the time of relapse since initial adjuvant therapy, prior treatment with CDK4/6 inhibitors in the adjuvant setting, nodal status, menopausal status, and geographic region [27]. Currently, the trial is actively recruiting patients.

Similarly to the EMBER-4 trial, after the results of the SERENA-2 trial [28,29], the CAMBRIA-1 trial is enrolling patients who underwent surgery and already received 2–5 years of prior endocrine therapy. Participants are assigned to continue their current standard endocrine therapy or switch to camizestrant at a daily dose of 75 mg. Stratification factors include patients’ risk profile, duration of prior standard endocrine therapy, menopausal status at randomization, and the option to receive CDK4/6 inhibitors for those deemed at high risk. Recruitment is still ongoing (NCT05774951).

Conversely, the CAMBRIA-2 trial has been designed to investigate camizestrant vs. standard endocrine therapy in patients with early breast cancer who did not receive adjuvant endocrine therapy. A total of 5500 patients who have undergone surgery but have not received any prior endocrine therapy will be randomized to receive camizestrant or standard endocrine therapy. Furthermore, patients who meet the criteria for CDK4/6 inhibitors are eligible to receive them as part of their treatment regimen. Stratification factors in CAMBRIA-2 include the risk of recurrence, menopausal status, and the utilization of CDK4/6 inhibitors (NCT05952557).

Adjuvant elacestrant is being tested in the TREAT ctDNA trial. Patients must have received at least 4 and a half years of endocrine therapy before enrollment and test positive for circulating tumor DNA (ctDNA) to be potentially enrolled in the trial (i.e., “ctDNA relapse” with no evidence of distant metastasis). Eligible patients are randomized to receive elacestrant or continue the previous endocrine therapy regimen when testing positive for ctDNA. Two hundred twenty patients will be enrolled in the phase. Recruitment for this trial is currently underway (NCT05512364).

Addressing the possibility of improving the tolerability of endocrine therapy and the compliance of the patients to complete adjuvant endocrine therapy, the phase III AMEERA-6 trial has been designed to investigate the use of amcenestrant compared to tamoxifen in patients who did not tolerate aromatase inhibitor [30].

Other trials are currently ongoing to expand the data of SERD in adjuvant setting (Table 1).

### Oral selective estrogen receptor degrader in the neoadjuvant setting and window of opportunity trials

The window of opportunity (WOO) trials are different from neoadjuvant trials due to the time frame in which patients receive an experimental drug, which usually in these trials is relatively short and between the cancer diagnosis and the start of the standard of care treatment.

Different trials in breast oncology have been designed including a WOO phase, for the numerous advantages, especially in terms of drug development and translational research (i.e., biomarkers identification) collecting baseline naive tumor samples after a short-term treatment. Treatment-naive tumors are biologically less heterogeneous than other cancer samples collected during the disease course, characterized by the emergence of resistance mechanisms under treatments’ selective pressure [31,32]. Suggested that a short-term change in proliferation, evaluated in terms of Ki-67 expression with a prespecified threshold at 10%, during 2 weeks of neoadjuvant endocrine therapy with either an aromatase inhibitor or tamoxifen, predicts the outcome of these patients [33]. Similarly, different WOO trials in breast cancer evaluated whether tumor response to neoadjuvant CDK4/6i plus aromatase inhibitor could somehow inform the response to the combination when administered in the adjuvant setting [34]. According to these results, different trials investigating neoadjuvant oral SERDs included a WOO phase. The coopERA trial is a randomized phase II study of neoadjuvant palbocilib plus giredestrant or anastrozole in postmenopausal patients with HR-positive/human epidermal growth factor 2 (HER2)-negative early breast cancer. In the WOO phase, 211 patients with tumor at least 1.5 cm and Ki67 at least 5% were randomly assigned to receive 2 weeks of giredestrant or anastrozole as monotherapy (days 1–14), the primary endpoint was met, since the Ki-67 change from baseline after the 2 weeks of monotherapy treatment was 75% with giredestrant and 67% with anastrozole (*P* = 0.043). A neoadjuvant phase of the same trial added palbociclib to ET in each arm for 16 weeks before surgery. A relative reduction in Ki-67 was observed in both arms but superior with giradestrant plus palbociclib (81 vs. 74%). No differences were observed in terms of overall response rate and pathological complete response rate between the two treatment arms [35]. Ongoing trials are testing giredestrant in the adjuvant setting [36,37].

Another preoperative WOO study is the EMBER-2 trial, evaluating imlunestrant in 58 postmenopausal patients with HR-positive/HER2-negative early breast cancer (stage I-III, T ≥1 cm, estrogen receptor >50%) randomly assigned to imlunestrant 400 mg *QD* or 800 mg *QD* for 15 days. Estrogen receptor expression changes represent the primary endpoint, while Ki-67 changes were among secondary objectives, along with safety and tolerability. Similar to giredestrant, imlunestrant was responsible for a decrease of the proliferation marker Ki-67, irrespective of the dose (-71% with imlunestrant 400 mg, -72% with imlunestrant 800 mg) [38].

As for the elacestrant, the ELIPSE trial recruited patients whose tumors have similar characteristics to those included in the coopERA and the EMBER-2 trials (T ≥1.5 cm, cN0 and Ki67 ≥10%), to evaluate whether 4 weeks of preoperative elacestrant can suppress Ki-67. Complete cell cycle arrest was the primary endpoint, achieved in 27% of patients, confirming a significant biological rationale for continuing testing elacestrant in early breast cancer [39].

In the AMEERA-4 trial, postmenopausal patients with HR-positive HER2-negative EBC were randomized in a 1 : 1:1 fashion to receive amcenestrant at two different doses (400 or 800 mg q.d.) or letrozole 2.5 mg q.d. for 14 days before surgery. The study closed early due to slow accrual with no formal compared results between the three arms. However, a decrease in the estrogen receptor H-score was observed in SERD arms (at both doses), but no difference in the Ki-67 expression [40].

Neoadjuvant camizestrant has been tested in the SERENA-3 study, which enrolled postmenopausal patients with HR-positive/HER2-negative early breast cancer. This trial was designed to evaluate the impact of 5–7 days of three different camizestrant doses (75, 150, or 300 mg once daily) on estrogen receptor expression (primary endpoint), progesterone receptor expression, and Ki-67 expression (both secondary endpoints, as safety and tolerability). After 12–15 days, Ki-67 score reduction was similar with 75 and 150 mg/day, supporting 75 mg/day of camizestrant as the optimal dose [41].

Of note, an arm of the adaptive I-SPY2 trial is evaluating neoadjuvant amcenestrant monotherapy (A) or combined with abemaciclib (AA), or with letrozole (AL), one in a selected population, defined as women or men with primary tumor at least 2.5 cm and MammaPrint low-risk, or MammaPrint high-risk but with nodal negative staging (NCT01042379).

It is worth noting that the data discussed are focused on postmenopausal women, however, as in the adjuvant setting, neoadjuvant trials enrolling premenopausal women are ongoing (Table 2) (e.g., the PREcoopERA and the PREMIERE trials).

**Table 2:** Neoadjuvant and WOO trials with oral selective estrogen receptor degrader

Drug	Trial name (NCT n.)	No. of patients	Design (phase)	Experimental arm	Control arm	Enrolled population	Primary endpoint(s)	Status
Elacestrant	SOLTI-1905 ELIPSE (NCT04797728)	23	Single group, open-label (Phase 0, WOO)	Elacestrant 400 mg PO, QD, 4 weeks (±2 days)	NA	Postmenopausal women, cT1c-3 (minimal 15 mm of largest diameter) cN0 cM0, ER+/HER2- breast cancer	Complete cell cycle arrest (Ki67 ≤ 2.7%) after 4 weeks (±2 days) of elacestrant therapy	Completed
	PremiÈRe (NCT05982093)	48	Randomized, open-label, WOO	Elacestrant vs. Elacestrant plus triptorelin	NA	Premenopausal women with early HR+/HER2- breast cancer (stage I-IIB)	Evaluate the biological activity of elacestrant ± OFS in premenopausal women with ER+/HER2- operable EBC [rate of CCCA determined by central assessment by IHC (% Ki-67 ≤ 2.7%) after 4 weeks of therapy]	Recruiting
Imlunestrant	EMBER-2 (NCT04647487)	90	Randomized, open-label, (Phase 1, WOO)	Imlunestrant PO (dose 1, dose 2, dose 3)	NA	Postmenopausal women, early-stage ER+/HER2-, stage I-III breast cancer	Change from baseline in ER expression	Completed
Camizestrant	SERENA-3 (NCT04588298)	135	Randomized, open-label parallel-group study (Phase 2)	Camizestrant (dose A, dose B, dose C)	NA	Postmenopausal women with ER+/HER2- early-stage breast cancer	Change from baseline in ER expression between pre and on-treatment tumor samples measured by IHC	Completed
Amcenestrant	AMEERA-4 (NCT04191382)	105	Randomized, window study (Phase 2)	Amcenestrant 400 mg or amcenestrant 200 mg	Letrozole 2.5 mg	Postmenopausal women with ER+/HER2- early operable breast cancer (stage I-III, excluding T4)	Percentage change from baseline in Ki-67 level at Day 15	Terminated
	I-SPY1 EO (NCT01042379)	5000	Randomized, open-label (Phase 2)	Amcenestrant 200 mg PO, QD ± abemaciclib/letrozole	NA	Women or men with primary tumor ≥ 2.5 cm and MammaPrint low-risk, or MammaPrint high-risk but with nodal negative staging ER+/HER2− breast cancer	pCR rate	Recruiting
Giredestrant	PREcoopERA (NCT05896566)	220	Randomized, open-label, WOO	Giredestrant vs. Giredestrant plus triptorelin	Anastrozole plus triptorelin	Premenopausal women with ER+/HER2- early operable breast cancer (stage I-III, excluding T4)	Change in Ki-67 (measured by IHC in central laboratory) between the pre and posttreatment tumor biopsy)	Recruiting
	Neo-AGILE (NCT06259929)	51	Single-arm, open-label (Phase 2)	Giredestrant plus abemaciclib	NA	Postmenopausal women, early-stage ER+/HER2- breast cancer	Efficacy of abemaciclib and giredestrant CCCA rate (defined as the proportion of patients with centrally assessed Ki67 scores ≤ 2.7%)	Not yet recruiting

CCCA, complete cell cycle arrest; ER, estrogen receptor; ET, endocrine therapy; HER2, human epidermal growth factor 2; IHC, immunohistochemistry; *N*, number; NA, not applicable; OFS, ovarian function suppression; PO, *per os*; QD, *quoque die*; WOO, window of opportunity.

## FUTURE PERSPECTIVES

It is still unknown whether employing oral SERDs in the (neo)adjuvant setting will significantly improve long-term outcomes of the patients. More evidence is needed to better understand if the second generation of oral SERD would play a role in the management of EBC. Early recurrence of HR+ EBC during or soon after the end of adjuvant endocrine therapy, in which SERDs may be potentially effective, have not yet been investigated. Moreover, combination strategies might improve the efficacy of oral SERD in the (neo)adjuvant setting.

Other then SERD, novel classes of ET are under investigation, like Selective Estrogen Receptor Covalent Antagonists (SERCAs), or PROteolysis Targeting Chimera (PROTAC) protein degraders, with preliminary data in the metastatic setting but no evidence in EBC.

## CONCLUSION

Optimizing the management of EBC remains a priority to increase the number of potentially cured patients. Oral SERDs can improve the compliance and quality of life of patients with EBC, having a different spectrum of adverse events than other endocrine therapy, although few data are currently available on efficacy.

The best timing and duration of SERD administration in (neo)adjuvant setting remain largely unknown. Specific biomarkers are needed to select and tailor patients that might benefit from (neo)adjuvant SERD treatment.

## Acknowledgements

*Author contributions: Conceptualization: P. Zagami; Methodology: all authors; Supervision: P. Zagami, G. Curigliano; Roles/Writing – original draft: all authors; Writing – review & editing: all authors.*

### Financial support and sponsorship

*None.*

### Conflicts of interest

*L.A.,. P.Z., and Gr.C.: none to declare; G.C.: Leadership: ESMO, European Society of Breast Cancer Specialists (EUSOMA), ESMO Open. Honoraria: Ellipses Pharma Consulting or Advisory Role: Roche/Genentech, Pfizer, Novartis, Lilly, FoundationMedicine, Bristol Myers Squibb, Samsung, AstraZeneca, Daichi Sankyo, Boehringer Ingelheim, GlaxoSmithKline, Seagen, Guardant Health, Veracyte, Celcuity, Hengrui Therapeutics, Menarini, Merck, Exact Sciences, Blueprint Medicines, Gilead Sciences. Speakers’ Bureau: Roche/Genentech, Novartis, Pfizer, Lilly, Foundation Medicine, Samsung, Daiichi Sankyo, Seagen, Menarini, Gilead Sciences, AstraZeneca, Exact Sciences. Research Funding: Merck (Inst). Travel, Accommodations, Expenses: Roche/Genentech, Pfizer, Daichii Sankyo, AstraZeneca. All COI are outside of this work.*

## REFERENCES AND RECOMMENDED READING

Papers of particular interest, published within the annual period of review, have been highlighted as:

▪ of special interest
▪▪ of outstanding interest
